# Optimizing the cryopreservation and post-thaw recovery of natural killer cells is critical for the success of off-the-shelf platforms

**DOI:** 10.3389/fimmu.2023.1304689

**Published:** 2023-12-15

**Authors:** Jennifer N. Saultz, Folashade Otegbeye

**Affiliations:** ^1^ Division of Hematology/Medical Oncology, Oregon Health and Science University, Portland, OR, United States; ^2^ Translational Science and Therapeutics Division, Fred Hutchinson Cancer Center, Seattle, WA, United States

**Keywords:** NK cells, allogeneic NK cell immunotherapy, NK cell cryopreservation, off-the-shelf application, thaw process

## Abstract

Natural killer (NK) cells are a promising allogeneic, off-the-shelf, cellular immunotherapy product. These cells can be manipulated ex vivo, genetically edited to enhance tumor targeting and expanded to produce large cell banks for multiple patient infusions. Therapeutic efficacy of these products depends on the recovery of viable and functional cells post-thaw. Post-thaw loss of viability and cytolytic activity results in large, and often variable, discrepancies between the intended cell dose (based on counts at cryopreservation) and the actual dose administered. Compared to their highly activated state in fresh culture, post-thaw NK cells demonstrate critical changes in cytokine production, cytotoxic activity, *in vivo* proliferation and migration. When these NK cells are introduced into the highly immunosuppressive tumor microenvironment, the functional changes induced by cryopreservation further limits the clinical potential of these products. This report will review the impact of cryopreservation on *ex vivo* expanded NK cells and outlines strategies described in published studies to recover post-thaw function.

## Introduction

Many cellular immunotherapy products (both commercially available and in clinical trials) require single patient product manufacturing streams from autologous donor sources. This individualized manufacturing approach requires significant coordination around patient care and manufacturing slots, often resulting in delays during which disease progression or other clinical deterioration can occur. Therefore, there is a critical need for readily available, safe and effective cellular therapies. Off-the-shelf products, such as allogeneic natural killer (NK) cell-based products and bispecific T cell engagers, can be made readily available without interrupting or delaying patient care. Although evolving, the T cell therapy field remains limited to autologous products due to the potential for graft versus host disease (GVHD) with allogeneic T cells. On the other hand, natural killer (NK) cells, which are not known to induce GVHD, are promising for developing off-the-shelf, allogeneic cellular immunotherapy products. Over the last 15 years, major advancements in the ability to activate and expand NK cells ex vivo have occurred allowing for broad use of these cells for various clinical applications (1–4). These manufacturing platforms require optimization of genetic manipulation, expansion and storage procedures, all of which have major implications on cellular function and viability. In this manuscript, we review the impact of cryopreservation on manipulated NK cells and explore strategies that have been associated with post-thaw recovery of viability and function.

Natural killer (NK) cells are lymphocytes which play a critical role in the control and elimination of virally infected and malignant cells (5). Because they are not dependent on tumor/tissue-specific antigen presentation, NK cells serve a vital role in innate and adaptive immune surveillance. The use of adoptive NK cellular therapy was pioneered from studies published in 2002 and 2005 by Ruggeri (6) and Miller (7) respectively. Their clinical findings showed that alloreactive NK cells can eliminate tumor cells by exploiting human leukocyte antigen (HLA) mismatches and/or mismatches between several HLA epitopes and their corresponding NK cell receptors known as killer cell immunoglobulin like receptors (KIR). Since they are not tumor-specific antigen restricted as in chimeric antigen receptor T (CART) cell therapy, NK cells have the innate ability to directly kill cancer cells without off-target toxicity. Their cytotoxic activity also does not drive the inflammatory syndromes seen with CART cell therapy such as cytokine release syndrome (CRS) and immune effector cell-associated neurotoxicity syndrome (ICANS). For all these reasons, along with the absence of GVHD induction in the allogeneic setting, NK cells are being increasingly investigated as optimal for off-the-shelf, allogeneic cellular therapy.

**Figure 1:**
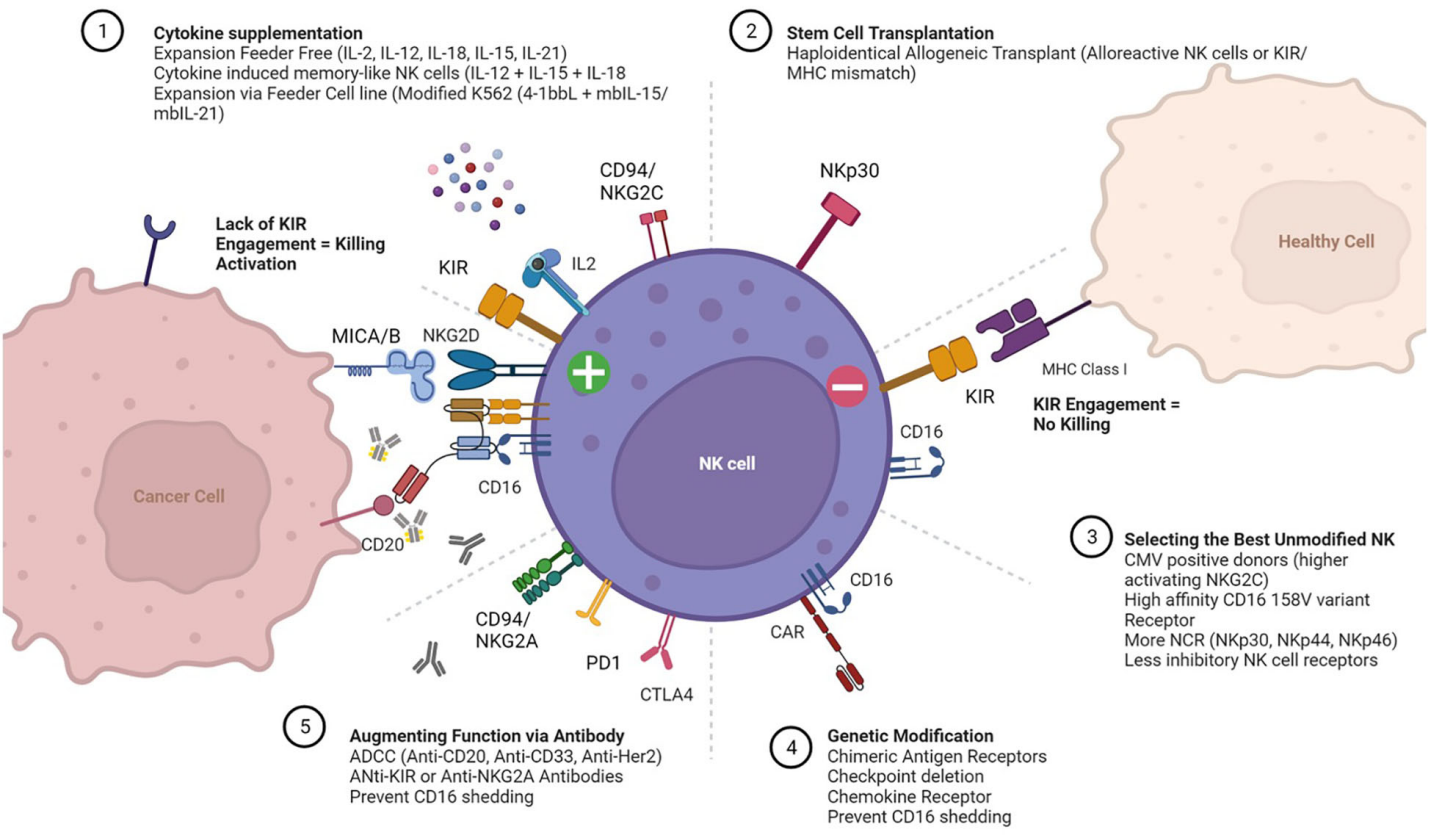
Expansion and Activation of Adoptive Natural Killer (NK) Cells. Cartoon depicting receptors that mediate interactions between NK cells (center), healthy cells (top right) and cancer cells (left). The more common approaches for (1) expanding NK cells *ex vivo* with cytokine supplementation; (2) post-transplant adoptive transfer; (3) selecting “optimal” NK cell donors; (4) *ex vivo* genetic modification of NK cells prior to expansion; and (5) administering agents to enhance NK cell function *in vivo* are also summarized.

NK cells have germline encoded receptors which dictate function. Killer-cell immunoglobulin-like receptors (KIRs) are transmembrane glycoproteins expressed on the plasma membrane of NK cells responsible for interacting with HLA Class I molecules expressed on all nucleated cell types. When HLA Class I is missing while an activating signal is present, the inhibitory KIR is not engaged and the NK cell is activated to kill (8). The interplay of these receptors, balancing inhibitory and activating signals determines cytolytic activity. The major receptors associated with activation include the natural cytotoxicity receptors (NCR) NKp30, NKp44, NKp46), as well as NKp80, NKG2D, FASL, CD155/CD112 and the heterodimer CD94/NKG2C (9). In addition, CD16 binds immunoglobulin Fc segment and augments antibody dependent cellular cytotoxicity (ADCC). Adoptively transferred, unmanipulated NK cells capitalize on these intrinsic properties to augment antibody therapy toward a particular cell antigen target such as CD20 in lymphoma (Rituximab) or HER2 in breast cancer (Herceptin). Even in the absence of genetic modification, adoptive NK cell function can be enhanced through the biased selection of “optimal” NK cell donors based on specific phenotypes. Examples of these phenotypes include CMV positive donors with high levels of activating CD94/NKG2C receptor (10); donors selected for the high affinity CD16 158 V variant receptor (11) and/or donors KIR mismatched for optimal alloreactivity (6). Various approaches to selecting NK cell donors, enhancing activation, and promoting expansion are summarized in **Figure 1**. In all these approaches, the critical functional NK cell receptors induced or activated require sustained expression and activity throughout the *ex vivo* manufacturing, storage and distribution of NK cell products.

A major limitation to the clinical application and scalability of NK cellular therapy has traditionally been both the limited number of NK cells in donor circulation (10% or less of total lymphocyte population) and inability to expand large numbers of NK cells for clinical use. In the last 15 years, significant improvements have occurred that allow for large scale expansion thus it is no longer a limitation in the field (12–14). NK cells can be obtained from multiple sources including cord blood and apheresis of allogeneic healthy donor peripheral blood mononuclear cells (PBMC). Once enriched (from PBMC) or differentiated (from cord blood), the isolated NK cells are expanded for further clinical applications. Expansion methods include co-culture with irradiated artificial antigen presenting cells (aAPCs) that present membrane bound (mb) gamma chain cytokines like IL-15 or IL-21 with or without co-stimulatory molecules (12–16). Culture conditions in these expansion platforms are supplemented with cytokines that promote robust proliferation and result in highly activated cells with enhanced cytotoxic activity. The success of these platforms has been broadened to include genetic manipulation of NK cells as with recent CD19-targeted CAR NK cells derived from cord blood sources (17). The high NK cell yields achieved can sufficiently generate multiple product aliquots for future patient infusions. *Ex vivo* expanded NK cell products have been infused in clinical trials of several malignancies including AML/MDS (15, 16, 18), colorectal (16), ovarian (19), pancreatic (20), breast (21), gastric (22), and neuroblastoma (23) with mixed results. As most NK cell products generated on these platforms are cryopreserved prior to administration, it is imperative to ensure adequate post-thaw recovery of planned cell doses, preserve their cytotoxic activity and ensure tumor migration capability.

### Changes induced by cryopreservation

Cryopreservation of NK cells, historically using freezing media containing 10–20% dimethyl sulfoxide (DMSO), has long been known to result in poor post-thaw viability and function. This effect is particularly seen in highly activated NK cells derived from *in vitro* manipulation or expansion cultures. Changes induced by cryopreservation on NK cell viability, immunophenotype, cytotoxic activity and pharmacokinetics are outlined below.

### Cell viability

While several studies have shown varying impact of cryopreservation, thaw and post-thaw processing techniques on the functional recovery of NK cells, viability is consistently and uniformly impaired over time. From several manufacturing qualification runs of NK cells expanded in 12-day co-culture with K562-mbIL15, Damodharan et al. demonstrated post-thaw viable NK cell recovery of 64% - 91%. This post-thaw recovery depreciated further over time with a viability decrease from a mean of 72% immediately post-thaw to 34% after 24 hours despite the addition of IL-2 supplementation in culture (12). A similar study of NK cells co-cultured with autologous feeder cells by Torelli et al. demonstrated 85% - 94% immediate post-thaw viability (24). Additionally, Lapteva et al. described donor-dependent difference in post-thaw recovery associated with length of cryopreservation (25). In their study, NK cells from all donors averaged 91% viability post-thaw after short-term storage but following 12 months of cryopreservation, post-thaw cell recovery ranged from 51 – 95%, highly dependent on the donor (25). Domogala et al. found that human AB serum with 10% DMSO was superior to further additions of cell culture media into the cryomedia for post-thaw recovery of NK cells derived from CD34+ cord blood cells (26). This study also showed uniformly poor recovery when cells were cryopreserved at low cell densities (44% at 10^6^/mL and 29% at 5 x 10^6^/mL).

Even in studies showing initial post-thaw viability similar to freshly harvested cells, such as the PM21-particle expanded healthy donor NK cells described by Oyer et al, viability decreased rapidly over time and could not be salvaged by IL-2 supplementation (27). For example, from qualification runs of 19 donor products utilizing this platform, there was a wide range of recovery (mean 73 ± 22%) after overnight incubation in media supplemented with 100U/mL IL2 (27). Similarly, Voshol et al. showed decrease in post-thaw viability to 50% during culture with or without IL-2 supplementation of culture media (28). Thus, viability is lost with cryopreservation even when cytokine supplementation is used. These findings all suggest that cell density, cryomedia composition, cryopreservation time and post thaw cytokine support all impact viability and need to be important process development considerations.

### Immunophenotype

Immunophenotype changes which impact NK cell function are common after cryopreservation. Damodharan et al. studied the effect of freeze thaw on NK cell ligands and found that post thaw activating receptor NKG2D was decreased on thawed NK cells compared to before cryopreservation (12). However, the natural cytotoxicity receptors (NCRs) NKp44, NKp46, and NKp30, which are also critical for activating NK cell mediated cytotoxicity, were not changed post-thaw. Importantly, KIR expression on NK cells was also unchanged between frozen and fresh NK cells suggesting that licensing/education of the infused NK cells is not impacted by cryopreservation. Cryopreservation however has been shown to potentially impact ADCC via cleavage of CD16 with a trend toward decreased expression of CD16 and NKG2D that was not statistically significant at both 1- and 16-hours post-thaw (27). Other activation markers were preserved. In a different study by Mark et al, the CD16+ population was found to decrease significantly after cryopreservation although this decrease did not correspond with change in function using CD107a expression as a surrogate suggesting that the NK cell present were still capable of degranulation upon target recognition (29). A hypothesis from this study is that cryopreservation induces cleavage of CD16 by the activation of matrix metalloproteinases. However further investigation is needed to determine if this activation is truly driving the decreased CD16 expression.

### Cytokine production

Cytokine production is critical for NK cell function and maintenance. Intrinsically, NK cells require secretion of interferon gamma (IFNγ), tumor necrosis factor alpha (TNFα) and granzyme B for anti-tumor response. The impact of cryopreservation on cytokine production by NK cells varies greatly between studies. Oyer et al. found no significant differences in the intracellular expression of IFNγ or the surface expression of the degranulation marker CD107a at both 1- and 16-hours post-thaw compared to fresh cells (27). TNFα expression decreased at 1h post-thaw compared to fresh cells but recovered after 16 hours of post-thaw rest. In comparison, Damodharan et al. found decrease in IFNγ expression but unchanged granzyme B post-thaw (12). In the same study, blocking STAT1 inhibition with Ruxolitinib, reduced interferon gamma production from fresh NK cells but had no significant effect on cryopreserved and thawed NK cells suggesting that the interferon gamma production in cryopreserved NK expanded NK cells is driven by factors other than IL-2 and IL15. Since Ruxolitinib is now FDA approved for the treatment of acute and chronic GVHD in post-transplant setting, it is critical to understand these functional differences.

### Cytolytic activity

While short-term storage up to 24 hours at 4°C has no impact on cytotoxicity (28) of NK cells compared to freshly harvested from culture, cytotoxicity is significantly impaired following cryopreservation. Freeze/thaw appears to have less effect on NK cell cytotoxic function when the cells are expanded in culture prior to cryopreservation and recovered with cytokine support in culture post-thaw. In the study by Damodharan et al., *in vitro* cell killing, assessed using live imaging, was observed to decrease over time post-thaw, with cytotoxicity greatest at 4 hours and negligible at ≥ 24 hours (12). The cytotoxicity decrease observed 24 hours post-thaw was likely related to loss of viability as the effector (E) to target (T) numbers were based on immediate post-thaw NK cell counts. Post-thaw NK cells in this study still demonstrated antibody-independent and dependent killing when used immediate post-thaw against various cell lines during 4-hr cytotoxicity assay. Mark C et al. also demonstrated decreased cytotoxicity in a chromium release assay which was more pronounced at lower E:T ratios of 2.5 – 5:1 (29).

Recovery of cytolytic function has been demonstrated as achievable by cytokine-supplemented culture conditions post-thaw. Lapteva et al., found that cryopreserved expanded NK cells were ineffective at lysing K562 cells at 20:1 E:T ratio despite excellent viability (91%) post thaw. Lysis of K562 targets at the same E:T ratio was however restored to an average of 79% when the post-thaw NK cells were rested overnight in complete SCGM medium supplemented with 10 U/mL IL-2 (25). Holubova et al. also found that cytotoxicity of K562 cells was most optimal with 24-hour re-stimulation of NK cells in IL-2 supplemented media with most significant difference noted at lower E:T ratios (5:1) (30). The timing of IL-2 re-stimulation also appears to be critical for functional recovery. Against a Burkitt cell line (Daudi cells), Voshol et al. showed that cell rest with post-thaw incubation for 24 to 48 hours prior to IL-2 stimulation led to recovered cytotoxicity compared to no IL-2 stimulation immediately post-thaw (28). At all E:T ratios, NK cells that were first rested in media for 24 hours prior to 100U/mL IL-2 stimulation had twice the cytotoxic activity against Daudi target cells compared to NK cells that were immediately cultured in IL-2 supplemented media for the 24-hour incubation. Of note, freshly thawed cells had no activity at 10 – 80:1 E:T ratios.

Perhaps reflective of pre-freeze activation, cryopreservation and thaw technique, cryopreserved PM21-NK cells that were thawed and rested showed no decrease in cytotoxicity when co-incubated with tumor cells at varying effector-to-target (NK:T) ratios compared to expansion-matched fresh NK cells (27). Over time however, cytotoxicity declined even for this cell product: when tested one-hour post-thaw without IL-2 rest, they became 21% less cytotoxic against K562 than the fresh cells. These findings were reproduced in *in vitro* kinetic live cell imaging assay at 1:1 E:T ratios against ovarian cancer cell line (SKOV-3), as well as in a 3D tumor model of ovarian and lung cancer cell spheroids. In an SKOV-3 xenograft, the same survival benefit of intraperitoneal NK cell administration was observed for fresh, 1h post-thaw or 16h post-thaw plus IL-2 rested NK cells at the same viable cell dose of 1 x 10^7^ for each group. This survival benefit of cryopreserved PM21-NK cells was replicated in a PDX murine xenograft model (27).

Other studies have shown that even when viability and *in vitro* cytotoxic function are preserved post-thaw, *in vivo* efficacy can still be impaired. Min et al. demonstrated preserved viability immediate, 24-hour and 48-hour post-thaw in large scale qualification runs of NK cell ex vivo expansion using irradiated autologous PBMC as feeder cells (14). The harvested NK cells were cryopreserved at 10^8^ cells per mL of cryomedia containing human serum albumin (HSA) with 50% RPMI1640, 25% Dextran-40 and 5% DMSO. As in previously reported studies, IL-2 supplemented culture had no impact on viability post-thaw. All conditions remained more than 90% viable immediate post-thaw with slight, not statistically significant, reduction at 24 hours, and no change at 48 hours. The natural cytotoxicity receptor NKp46 was the only receptor (activating or inhibitory) shown to significantly decrease post-thaw. Immediate post-thaw cytotoxicity against K-562 cells in a 4-hour assay and cytokine secretion *in vitro* remained the same as for fresh cells. Cytotoxicity was unchanged at 24 and 48 hours, with or without IL-2 stimulation. The only *in vitro* difference in this study was that at lower E:T ratios (3:1) *in vitro*, ADCC activity of post-thaw NK cells using Rituximab was reduced versus fresh, but no different at 10:1 E: T. Interestingly, despite the overall preserved *in vitro* function, *in vivo* antitumor inhibition was decreased using the same cell dose of fresh and post-thaw NK cells. This difference was only overcome, and antitumor inhibition promoted, by administering twice as many post-thaw NK cells as fresh. Post-thaw NK cells were however also able to mediate *in vivo* ADCC in combination with Rituximab in a murine model (14).

### *In vivo* kinetics

The discrepancy between salvageable cytotoxic function *in vitro* and limited efficacy *in vivo* of post-thaw NK cells compared to fresh cells suggests that other factors are at play. In a Phase I study of third-party, HLA-disparate adult donor NK cells expanded using OCI-AML-mbIL21 feeder cells and stored as cell banks for multiple patients, Otegbeye et al. showed peak NK cell donor chimerism at 7 days (4 – 20%) and persistence in circulation for up to 28 days (1 – 3% chimerism) after infusion (16). Of note, no exogenous cytokines were administered in that clinical trial. This persistence profile is similar to what is reported for fresh NK cell infusion studies (7) suggesting that distribution in circulation is not significantly impacted by freeze-thaw.

Cryopreservation may reduce NK cell motility such that they cannot make contact with target cells beyond their immediate neighbors. In a study of the NK cell line NK92 as well as NK cells derived from healthy donor PBMCs then expanded in 14-day culture with K562-mbIL15-41BBL feeder, Mark et al. (29) showed impairment of NK cell mobility by cryopreservation. Using a 3D collagen gel matrix, six-fold reduction in motile NK cells through the matrix was observed for post-thaw cells compared with fresh culture in time-lapse imaging (29). This impaired motility was not recovered by post-thaw culture up to 48 hours in the absence of IL2. When the gel matrix was also embedded with K562 cells as targets, the post-thaw NK cell fraction that retained motility also exhibited similar cytotoxic function as seen for fresh cells. Overall cytotoxicity of total post-thaw NK cells (motile and immotile) in this matrix model was decreased by a factor equivalent to the reduction in motility compared with fresh cells. These findings point to a critical limitation of NK cell therapy using cryopreserved cells in solid tumor environments where tumor infiltration is necessary for treatment efficacy.

### Cryopreservation and processing techniques associated with sustained post-thaw recovery

A summary of clinical or large-scale Good Manufacturing Practice (GMP) cryopreservation and thaw techniques in published literature is outlined in **Table 1**. Different studies have employed a variety of approaches to cell concentration, freezing media composition, thaw techniques (diluent composition, dilution factor) and post-thaw recovery using culture media supplemented with varying concentrations of IL-2. The use of lower DMSO concentrations (5%) and controlled rate freezing programs appear to improve post-thaw recovery, function and *in vivo* kinetics (14, 16). As previously discussed, while IL-2 supplemented culture post-thaw does not prevent the loss of viable cells, functional recovery is achieved to varying degrees. Post-thaw processing also provides an opportunity to ensure the desired cell dose is administered.

**Table 1:** Summary of NK cell formulation, cryopreservation and thaw procedures in published studies of clinical and GMP-qualification production.

Reference	Cell concentration (# of products studied)	Freeze Processing	Thaw Processing
Damodharan SN et al. (12)	N/A (N = 5)	Cryomedia: 50% Plasmalyte, 40% hAB serum, 10% DMSO Settings: CRF cooling 3°C/min till -180°C; vapor phase LN2	Thaw: 37°C water bath x 4-5min; product diluted with 10% dextran 40, then 5% HSA at 2:1:1 respectively; equilibration then further 10% dextran 40 dilution before centrifugation (2200rpm for 15 min at 10°C), then reformulation in 60% Plasmalyte, 40% HSA
Torelli GF et al. (24)	2 x 10^7^/mL (N = 4)	Cryomedia: 5% HSA + 10% DMSO. Settings: CRF Program N/A. 2mL vials, vapor phase LN2	N/A
Lapteva N et al. (25)	2 x 10^7^/mL (N = 5)	Cryomedia: HSA (Flexbumin) + 10% DMSO, 40% Hanks’ Balanced Salt Solution. Cryostore bags. Settings: Cryomed 7454 freezing profile	Post thaw: either immediate post-thaw or resting overnight in complete SCGM medium with 10U/mL IL2
Otegbeye F et al. (16)	5 x 10^7^/mL (N = 9)	Cryomedia: HSA + 5% DMSO	First cell dose level overnight thaw of 1.5 – 2 times intended dose and rest in 1000U/mL IL2 supplemented media, then next day formulation of cell dose for infusion. For 2^nd^ and 3^rd^ dose level, same day thaw of 1.5 times intended dose, then fresh formulation of cell dose infused.
Min B et al. (14)	1 x 10^8^/mL (N = 16)	Cryomedia: RPMI1640 50%, Albumin 20%, Dextran-40 25%, DMSO 5% Settings: CRF	37°C water bath then diluted with RPMI1640 + FBS 10%
Domogala et al. (26)	1 – 5 x 10^6^/mL (N = 26)	Cryomedia: hAB serum + 10% DMSO *vs* DMEM + AB serum 10% + DMSO 10%	1:10 dilution in media
Holubova et al. (30)	2 x 10^7^/mL (N = 14)	Cryomedia: PBS + 10% albumin + 10% DMSO.	Post-thaw maintained with or without IL2 1000 IU/mL for up to 24h.
Voshol H et al. (28)	0.5 – 1 x 10^7^/mL (N = 6)	Cryomedia: Cell suspension in media (RPMI1640) cooled on ice then equal vol. of freezing medium (RPMI 50%, FCS 30%, DMSO 20%) added slowly over 5 – 10 minutes. Settings: Frozen vials stored at -80 (not CRF)	Diluted 1:10 by dropwise addition of room temp medium, then media wash x 2. Resuspended for fresh analysis versus recovered with medium, 20% FCS +/- 100U/mL IL2, incubated at 37°C
Mark C et al. (29)	2.5 – 5 x 10^6^/mL (N = 24)	Cryomedia: HSA with 12.5% DMSO and 4% glucose Settings: Freezing in cryovials at 1mL/vial	Rapid thaw in 37°C bath until visible chip, then dropwise transfer into 10mL (10x dilution) of warmed (37oC) media
Oyer JL et al. (27)	1 – 2.5 x 10^7^/mL (N = 19)	Cryomedia 1: 50% RPMI, 40% FBS, 10% DMSO (for *in vivo*) Cryomedia 2: FBS-free, commercial cryomedia (for *in vitro*) Settings: Mr. Frosty in -80 overnight, then liquid nitrogen	37°C water bath, dropwise 5-fold dilution in RPMI with 10% FBS. Overnight thaw and rest at 1 x 10^6^/mL in RPMI + 10% FBS + 100U/mL IL2 x 16 hours.

CRF, Controlled Rate Freezing; DMSO, dimethyl sulfoxide; FBS, fetal bovine serum; FCS, fetal calf serum; HSA, Human Serum Albumin 25%; hAB serum, human AB serum; IL2, Interleukin-2; N/A, not available; PBS, Phosphate buffered saline; RPMI 1640, Roswell Park Memorial Institute 1640 medium; SCGM, stromal cell growth medium.

## Conclusion

There is ongoing research into approaches to replicate the preclinical efficacy of NK cells in clinical models. These strategies include use of small molecules or genetic modification to ensure auto/paracrine cytokine (IL-15) support *in vivo*, enhance NK cell infiltration and function in the tumor micro-environment, block inhibitory receptors, augment antigen recognition, and enhance activating signals. As these strategies evolve, the need to ensure that NK cells produced for off-the-shelf use maintain function and have minimal viability losses following cryopreservation will become even more critical. There is also a need to ensure that the phenotype induced by genetic or other manipulations to the NK cells during their primary culture are retained following cryopreservation. A study of NK cells complexed with bispecific cell engagers prior to 12-hour cryopreservation at -80°C showed that there was retained targeted cytolytic activity compared to non-cryopreserved cells (31).

The effects of cryopreservation on NK cell recovery and function appears to vary depending on cell of origin and the type of manipulation the cells were subjected to prior to freezing. While functional recovery appears achievable post-thaw by resting in media supplemented by IL-2, the progressive loss of viable cells is unchanged. This has significant implications for cell dose selection when based on cell count prior to cryopreservation. As such, early clinical trials of NK cell therapy focused on administration of cells harvested fresh from primary culture. This model, in addition to having limited scalability due to the required single product manufacturing from each donor, is also inconsistent with all the previously outlined benefits of an off-the-shelf cell therapy approach. The individualized manufacturing also results in variability between products administered to each patient, thereby limiting generalizability of product potency and treatment efficacy.

To generate predictable and reliable products, several clinical trials have conducted large-scale manufacturing, cryopreserved the harvested NK cells, then performed brief (overnight) cultures of post-thaw cells to re-activate them before administration to the study subjects. The common gamma chain receptor cytokines, particularly IL-2, which are critical for NK cell expansion and function are often used to supplement the post-thaw culture conditions. Other cytokines used to activate NK cells in these conditions include IL-12, IL-15, IL-18 and IL-21. This post-thaw recovery approach, while more amenable to allogeneic, off-the-shelf administration, does require additional post-thaw processing done in GMP-compliant facilities. This results in an added layer of complexity to off-the-shelf use and additional cost for each product infusion. While facility expertise in processing could be simplified by adopting closed and automated systems for thaw, incubation and formulation, appropriate quality control expertise for on-site release would still be required.

In summary, as the science of manipulating NK cells to generate potent immunotherapy products continually evolves, achieving the therapeutic potential and feasibility of off-the-shelf NK cell therapy will require optimization of cryopreservation and thaw techniques to ensure adequate functional recovery.

## Data availability statement

The original contributions presented in the study are included in the article/supplementary material. Further inquiries can be directed to the corresponding author.

## Author contributions

JS: Data curation, Writing – original draft, Writing – review & editing. FO: Conceptualization, Data curation, Supervision, Writing – original draft, Writing – review & editing, Methodology.
